# Inadequate Functional Health Literacy in Spanish as a Barrier to Cervical Cancer Screening Among Immigrant Latinas in New York City

**Published:** 2004-09-15

**Authors:** Samantha Garbers, Mary Ann Chiasson

**Affiliations:** Medical & Health Research Association of New York City, Inc; Medical & Health Research Association of New York City, New York, NY

## Abstract

**Objective:**

The objective of this study was to examine the association between inadequate functional health literacy in Spanish among low-income Latinas aged 40 and older and cervical cancer screening knowledge and behavior.

**Methods:**

Spanish-speaking Latinas aged 40–78 of various nationalities (n = 205) participated in a study that included a survey on cervical cancer knowledge and behavior administered in Spanish and the Spanish version of the Test of Functional Health Literacy in Adults.

**Results:**

Compared to those with adequate and marginal health literacy, women with inadequate functional health literacy in Spanish were significantly less likely to have ever had a Papanicolaou (Pap) test (odds ratio, 0.12; 95% confidence interval [CI], 0.04-0.37) or in the last three years (odds ratio, 0.35; 95% CI, 0.18-0.68) and were significantly more likely to have had their last Pap test at a local public hospital (odds ratio, 2.43; 95% CI, 1.18-4.97). Even when controlling for other factors, women with inadequate health literacy were 16.7 times less likely (adjusted odds ratio, 0.06; 95% CI, 0.01-0.55) to have ever had a Pap test.

**Conclusion:**

Almost half of the population we studied will have difficulty interpreting written medical materials, even in Spanish. When developing efforts to reach women who have not been screened, programs and service providers need to be aware that the women most in need of information about screening may be more likely to be unable to read any written materials provided to them, regardless of the language or level of simplicity of the materials. Programs and strategies need to be implemented to increase screening prevalence and to minimize the identified gaps in regular screening for Latinas who have low health literacy.

## Introduction

Health literacy has been defined as "the degree to which individuals have the capacity to obtain, process, and understand basic health information and services needed to make appropriate health decisions" (1). Improving health literacy has been added as a *Healthy People 2010* objective (2), and two recent reports by the Institute of Medicine and the Agency for Healthcare Research and Quality summarize the data regarding the prevalence of low health literacy and its relationship to health care quality, use, outcomes, and disparities (3,4). Despite these findings, health care providers are often unaware of the health literacy skills of their patients (5–7).

While there has not been a large-scale study representative of the U.S. population examining health literacy per se, the 1992 National Adult Literacy Survey indicated that 40–44 million Americans demonstrated skills in the lowest level of literacy proficiency in three scales (prose, document, and quantitative) (8). Some populations are more likely to have lower literacy skills, including the elderly, non-native English speakers, those with limited formal education, immigrants, and those with low incomes (8-11). In New York City alone, according to the 2000 U.S. Census, more than 1.22 million foreign-born residents arrived in the United States in the previous 10 years (12).

There are numerous barriers to effective cervical cancer screening, particularly for populations with low health literacy: screening recommendations for cervical cancer are complex (13,14), and educational materials are often written at reading levels that are inappropriately high for most of the population (15,16). For women who are native Spanish speakers, there are even fewer appropriate materials. Cervical cancer is preventable and treatable if detected early, yet in 2004 there will be an estimated 10,520 new cases of invasive cervical cancer diagnosed in the United States, and 3900 cervical cancer deaths (17). Case-control studies have found that the risk of developing invasive cervical cancer is three to 10 times greater in women who have not been screened (18). SEER (Surveillance, Epidemiology, and End Results) data show that cervical cancer incidence among Latinas aged 30 years and older is almost two times higher than the rate among non-Hispanic white women (19), which likely reflects disparities in screening prevalence (20-22). Low income, educational attainment, acculturation, and literacy may contribute to lower rates of screening (20,23-25).

As the populations at risk for low health literacy continue to increase both in New York City and in the United States, and the ethnic disparities in cervical cancer incidence widen, reducing the health-literacy–related barriers to cervical cancer screening and appropriate follow-up becomes an even more serious public health concern. Building on a previously published study that found an association between health literacy (in English) and Papanicolaou (Pap) test knowledge among a multiethnic group of young women (7), our study examined the independent association between functional health literacy in Spanish among low-income Latinas aged 40 and older and cervical cancer screening knowledge and behavior.

## Methods

Women aged 40 and older were recruited for the study through their younger female relatives. On approximately three recruitment days each week, from November 2002 to July 2003, all women who were awaiting appointments for prenatal care and family planning services at two MIC-Women's Health Services Centers operated by Medical and Health Research Association of New York City, Inc (MHRA) were approached in the waiting room. In 2003, the two clinic recruitment sites had 1879 visits from new prenatal and family planning patients. For women who self-identified as Latina or Hispanic, were aged 18 or older, and who had a female relative aged 40 or older living in the New York City area, the interviewer described the study, provided a flyer, and asked for the client's written informed consent to be contacted later to obtain the names and contact information of relatives aged 40 and older. A total of 1205 young women were approached in the centers: 936 did not fit the above listed eligibility criteria for referring participants, 27 were eligible but refused to refer participants, and 242 agreed to refer a participant. Older relatives were eligible to participate in the study if they self-identified as Latina or Hispanic, were aged 40 or over, and spoke Spanish as their primary language. Of the 242 women contacted for the study, 25 (10%) refused participation and seven (3%) were ineligible (two spoke English as a first language, five were under age 40). A total of 210 Latina women of various nationalities, ranging in age from 40–78, consented to participate in the study. Five participants completed the survey but refused participation in the Test of Functional Health Literacy in Adults (TOFHLA-S), a screening instrument in Spanish that has been used in several settings to identify patients with low functional health literacy; these women were not included in the analysis, leaving a final sample size of 205. The study was approved by MHRA's Institutional Review Board.

The interviews were administered in Spanish in participants' homes by a trained and experienced bilingual interviewer. Participation in the study included written informed consent, administration in Spanish of a 20-minute survey on cervical cancer screening knowledge and behavior, administration of the Spanish version of the TOFHLA-S and, for a randomly selected subset of 10% of participants, medical record release for validation of the most recent Pap test. All materials, including the consent form and survey, were written in Spanish and were read aloud to all participants. Materials were developed using simple words and short sentences. The informed consent (158 words) had an average of 14 words per sentence and average of 2.1 syllables per word. The survey, which included 36 questions, had an average of 10 words per sentence and 1.9 syllables per word. To facilitate recall and to reduce inconsistencies in reporting, the survey was developed using cognitive interviewing techniques in which participants were asked to verbalize their thought processes as they completed the survey (26). The survey was developed for the purposes of this study; several of the survey questions were adapted from a previous study on breast cancer screening knowledge and behavior (27). The survey was developed in English, translated to Spanish, and back-translated for review. Survey items included demographic information, most recent visits to health care providers, and detailed information on the most recent Pap test, from appointment making through follow-up. Open-ended questions asked about the purpose of a Pap test, how a Pap test is performed, and knowledge of risk factors for cervical cancer. The survey asked participants about the Pap test provider (type of site where the participant had her last Pap test [e.g., hospital, clinic, private physician office] and nation of provider [United States or native country]). Follow-up measures included whether the participant received her results, how she got results (e.g., postcard, phone call, visit to the provider), whether she was asked to return for a repeat Pap test, and whether she obtained her last two Pap tests in the same place.

Participants completed the TOFHLA-S. The English version of the TOFHLA has been tested for concurrent validity with other standardized literacy tests (28,29), but because there are no Spanish versions of the other standardized tests (REALM and WRAT-R) (30), concurrent validity with TOFHLA-S has not been measured. The TOFHLA-S includes both reading comprehension (employing a modified Cloze procedure) and numeracy sections. The results of the test yield a score from 0–100 that includes equal contributions from each section. The test takes up to 22 minutes to administer. The TOFHLA-S score is categorized into three levels: those with inadequate functional health literacy (TOFHLA score 0–59) are unable to read and interpret health texts, those with marginal functional health literacy (TOFHLA score 60–74) have difficulty reading and interpreting health texts, and those with adequate functional health literacy (TOFHLA score 75–100) can read and interpret most health texts.

Data analysis was performed using SPSS statistical software version 9.0 (SPSS, Inc, Chicago, Ill). Statistical differences in the frequencies of demographic characteristics, health care access (three dimensions: having any health insurance, having a regular source of care, and visiting any provider in the last 12 months), and Pap test knowledge, behavior, and follow-up by functional health literacy groups were assessed by chi-square tests for categorical variables and analysis of variance for continuous variables. Bivariate odds ratios and 95% confidence intervals were calculated for functional health literacy and demographic variables and Pap test behaviors. Using logistic regression, adjusted odds ratios and 95% confidence intervals were calculated for ever having a Pap test and having one in the last three years as the dependent variables, adjusting for characteristics associated with screening behavior (having a source of care [no {ref}/yes], having any health insurance [no {ref}/yes], age [40–49 {ref}, 50-59, 60 or older]) and those known to be associated with literacy (8) (years in the United States [0–14 years {ref}, 15 or more years {dichotomized at the median value}], education [elementary or less {ref}, some high school or more], and TOFHLA-S score [adequate {ref}, marginal, inadequate]) (31). A second logistic regression model separated women with inadequate functional health literacy into two groups: those who scored 1 or above, and those who scored 0 (unable to read any words).

## Results

**Figure 1:**
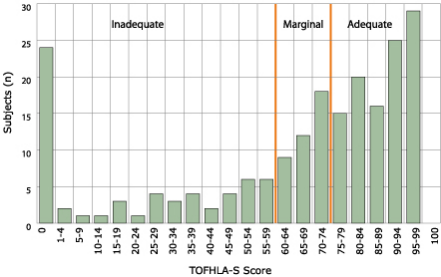
Distribution of scores in functional health literacy as determined by Spanish version of Test of Functional Health Literacy in Adults (TOFHLA-S) among Latinas aged 40 and older in New York City, November 2002–July 2003 (N = 205).

Table 1 presents sociodemographic and health information for the 205 women in the sample. More than half the women had elementary school education or less, and 95% were foreign-born. Access to care was low, according to three measures studied: 58% had no health insurance, 41% had no regular source of health care, and 22% had not visited any doctor in the last year. Almost all of the women interviewed had heard of a Pap test, and 92% had ever had a Pap test. While 75% could identify (in an open-ended question) that the purpose of a Pap test was to detect cancer, only three women specified cervical cancer. As illustrated in Figure 1, scores on the TOFHLA-S indicated a population with low health literacy levels in Spanish: 30% had inadequate health literacy in Spanish, 19% marginal, and 51% adequate. Twenty-four women (12%) were unable to read any words (TOFHLA-S score of 0). Significant differences were found by functional health literacy level on sociodemographic variables (except birthplace), knowledge of cervical cancer, and cervical cancer screening behavior. No differences were found in terms of access to health care: those with adequate health literacy were no more likely to have health insurance, a regular source of care, or to have visited any health care provider in the last year.

**Figure 2:**
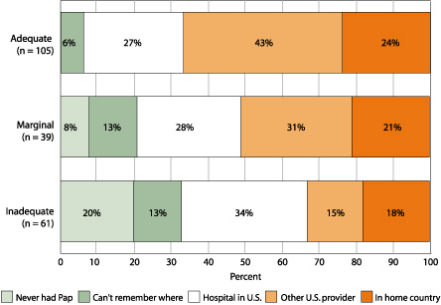
Location of most recent Papanicolaou (Pap) test by functional health literacy level: survey results among Latinas aged 40 and older in New York City, November 2002–July 2003. Literacy levels determined by Spanish version of Test of Functional Health Literacy in Adults (TOFHLA-S).

Although more than three quarters of the women had had a Pap test in the last three years, this population faced significant barriers to effective cervical cancer screening and follow-up, regardless of literacy level: more than 14% did not receive the results from their most recent Pap test, 10% could not remember where they had their last Pap test, 29% did not have their last two Pap tests in the same place, and 26% had their last Pap test in their native country. Figure 2 illustrates the location of most recent Pap test by literacy level. Compared to those with adequate and marginal health literacy, women with inadequate health literacy were significantly less likely to have ever had a Pap test (odds ratio [OR], 0.12; 95% CI, 0.04-0.37) or in the last three years (OR, 0.35; 95% CI, 0.18-0.68), and were significantly more likely to have their last Pap test at a local public hospital (OR, 2.43; 95% CI, 1.18-4.97; excluding those who had never had a Pap test or who could not remember where they had their last Pap test).

The goal of the study was to evaluate an independent association of functional health literacy in Spanish on Pap test behavior, taking into account factors known to be associated both with lower prevalence of screening and lower levels of literacy. Table 2 illustrates both the crude and adjusted odds ratios for ever having a Pap test and having one within the last three years. After adjusting for source of care, health insurance status, age, years in the United States, and education, women with inadequate functional health literacy in Spanish (compared to women with adequate health literacy) were 16.7 times less likely to have ever had a Pap test (adjusted OR, 0.06; 95% CI, 0.01-0.55). In the adjusted model, only having a source of health care was found to predict having a Pap test in the last three years (Adjusted OR, 3.67; 95% CI, 1.45-9.27).

Women who were unable to read any words (comprising 12% of the sample, and 39% of those with inadequate health literacy) were significantly different from women who had inadequate health literacy as measured by the TOFHLA-S but were able to read (data not shown in table). Compared to the rest of the sample, women who were unable to read any words were more recently arrived in the United States (75% in the United States less than 15 years, compared with 47%, chi-square = 6.54, *P* = .009), were significantly older (mean age 61.1 versus 49.6, ANOVA F = 38.96, *P* < .001), and had fewer years of schooling (95.8% with elementary education or less, compared to 44.2%, chi-square = 22.99, *P* < .001). A second logistic regression (data not shown in table) was conducted in which health literacy was divided into four strata; women who had inadequate health literacy but scored higher than 1 on the TOFHLA-S were categorized separately from those who scored 0 (were unable to read any words), although this is not a distinction that is made by the developers of the TOFHLA. In this analysis (with women with adequate health literacy as the referent group), after adjustment for the same variables as above, women who could not read any words were four times less likely (adjusted OR, 0.24; 95% CI, 0.07-0.85) to have had a recent Pap test, but no difference was found for women who had inadequate health literacy but scored higher than 1 on the TOFHLA-S.

## Discussion

Previous studies of literacy and functional health literacy in the United States have focused on populations' abilities to negotiate written information in English. As Latino populations in the United States continue to grow, exchanges of health care information will increasingly be provided in Spanish: the 2000 U.S. Census revealed that 28.1 million Americans speak Spanish, with only half reporting that they fluently speak English. However, our study suggests that taking the next step in addressing a health information gap — making materials available in Spanish — may not be adequate. Almost half of the women we studied will have difficulty interpreting written medical materials, even if the materials are made available in Spanish.

Because of low access to care in addition to low levels of functional health literacy, the study population faces significant barriers in obtaining effective cervical cancer screening. Our study adds to previous findings by Lindau et al, in which literacy (in English) was the only factor independently associated with knowledge of cervical cancer screening, even when controlling for age, education, ethnicity, employment, and insurance (7). In our study, low levels of functional health literacy in Spanish were strongly inversely associated with ever having a Pap test. Even when controlling for other factors (including age, educational level, having a source of care, having health insurance, and years in the United States) women with inadequate functional health literacy in Spanish were 16.7 times less likely to have ever had a Pap test.

Cervical cancer is preventable and curable if detected early. While the proportion of women we studied who had ever had a Pap test was high (92%), it is lower than the *Healthy People 2010* objective of 97% (2). Appropriate, regular screening and follow-up are essential to reduce the identified cervical cancer mortality and incidence gaps between Hispanics and non-Hispanic whites (32). For the outcome of clinical importance — having a recent (in the last three years) Pap test — the logistic regression revealed no independent association with functional health literacy level. A second regression model, however, separating women who could not read any words, revealed a strong association with having a recent Pap test.

The TOFHLA-S, used to measure health literacy in Spanish, does not distinguish between those who have inadequate health literacy and those who are unable to read any words. While the questions in the numeracy section are read aloud, the participant must be able to read both the prompts in the numeracy section and the reading comprehension section. Women with lower literacy skills may have been more likely to refuse participation because of the actual or perceived literacy burden of the study, resulting in a study population that underrepresents women with inadequate functional health literacy. It should be stressed that this study measured functional health literacy in Spanish, the primary language of participants. We did not measure participants' functional health literacy in English, which would allow greater comparability with previous studies that have examined associations between health literacy in English and health care knowledge and behavior. An unknown proportion of the women we studied who were found to have adequate functional health literacy in Spanish would not have adequate functional health literacy in English, the primary language in which health care services and education are provided in the United States.

This study had some other limitations. Previous research suggests that the prevalence of cervical cancer screening may be overreported (33,34). Self reporting, as our validation efforts confirmed, consistently results in overreporting of the prevalence of Pap testing (35,36). Because of the small sample size, the study lacked the power to detect significant effects of some characteristics that may also contribute to cervical cancer screening behavior, including nationality, years in the United States, and age. This analysis focused on Latina immigrant women in New York City whose primary language is Spanish. Caution should be used in applying the findings to other ethnic populations, to women living in other areas, or to Latinas whose primary language is English. Finally, the study examined the association between functional health literacy and cervical cancer knowledge and behaviors; however, the scope of the study did not include measurement of the complex relationships between screening knowledge and behavior.

Our findings are of particular importance to health care providers and screening programs that serve low-income, immigrant Latina communities. In the communities we studied, women with low functional health literacy were more likely to obtain their care at local public hospitals. Our study confirmed previous findings that women with low health literacy were no less likely to have a regular source of care and to have had a visit to a provider in the last year (37). Programs and strategies need to be implemented to increase screening prevalence and to minimize the identified gaps in follow-up for all patients. Patient/provider exchanges of all kinds (including those relating to cervical cancer screening) currently rely on the exchange of written information, including educational brochures, prescriptions, test results, and referrals for follow-up. Screening programs and service providers, when developing efforts to reach women who have not been screened for cervical cancer, need to be aware that the women most in need of information about screening may be more likely to be unable to read any written materials provided to them, regardless of the language or simplicity of the materials. Increasing cervical cancer screening rates and improving follow-up among Latinas with low functional health literacy will require creative solutions to convey information without relying on written materials. Providers face the added challenge that individuals with low functional health literacy may also have difficulties with oral communication with providers (38). The evidence on the effectiveness of interventions using innovative approaches such as videotapes is still emerging (4).

## Figures and Tables

**Table 1:** Demographic and Health Care Characteristics by Functional Health Literacy Level in Spanish: Survey Results Among Latinas Aged 40 and Older (n = 205), New York City, November 2002–July 2003^a^

		**Health Literacy Level in Spanish as Measured by Test of Functional Health Literacy in Adults (TOFHLA-S)**		

**Characteristic**	**All Women** (n = 205), No. (%)	**Inadequate** (n = 61), No. (%)	**Marginal** (n = 39), No. (%)	**Adequate** (n = 105), No. (%)

**Sociodemographic^b^**				

Age (years, mean)	51.0	55.3	51.4	48.3
Years in United States (mean)	17.9	13.8	21.0	19.1

**Birthplace** c				

United States (not Puerto Rico)	11 (5.4)	0 (0)	2 (5.1)	9 (8.7)
Mexico	25 (12.3)	10 (16.4)	4 (10.3)	11 (10.6)
South America	84 (41.2)	26 (42.6)	16 (41.0)	42 (40.4)
Dominican Republic	39 (19.1)	11 (18.0)	7 (17.9)	21 (20.2)
Puerto Rico	28 (13.7)	7 (11.5)	9 (23.1)	12 (11.5)
Central America	12 (5.9)	4 (6.6)	1 (2.6)	7 (6.7)
Other	5 (2.5)	3 (4.9)	0 (0)	2 (1.9)

**Level of education** d				

No formal education	12 (5.9)	12 (19.7)	0 (0)	0 (0)
Elementary school only	91 (44.4)	39 (63.9)	22 (56.4)	30 (28.6)
Some high school	38 (18.5)	8 (13.1)	5 (12.8)	25 (23.8)
High school graduate or more	64 (31.2)	2 (3.3)	12 (30.8)	50 (47.6)

**Marital status** e				

Married or living as married	100 (49.5)	26 (43.4)	17 (44.8)	57 (54.8)
Never married	27 (13.4)	6 (10.0)	7 (18.4)	14 (13.5)
Separated/divorced or widowed	75 (37.1)	28 (46.6)	14 (36.9)	33 (31.7)

**Health care** c				

No source of health care	83 (40.5)	27 (44.3)	14 (36.8)	42 (40.0)
No visit to health care provider in last year	45 (22.0)	16 (26.2)	4 (10.3)	25 (23.8)

**Insurance coverage** c				

No insurance	118 (57.8)	37 (60.7)	18 (47.4)	63 (60.0)
Medicaid and/or Medicare	66 (32.3)	20 (32.8)	18 (47.4)	28 (26.7)
Private insurance	20 (9.8)	4 (6.6)	2 (5.3)	14 (13.3)

**Knowledge**				

Ever heard of a pelvic exam^f^	198 (96.6)	56 (91.8)	38 (97.4)	104 (99.0)
Ever heard of a Pap test^d^	193 (94.1)	50 (82.0)	38 (97.4)	105 (100.0)
Can describe how a Pap test is performed^d^	162 (79.4)	35 (57.4)	31 (81.6)	96 (91.4)
Could specify that Pap was to detect cancer^f^	153 (74.6)	37 (60.7)	31 (79.5)	85 (81.0)
Ever heard of a mammogram^c^	154 (75.1)	47 (77.0)	29 74.4)	78 (74.3)

**Behavior**				

Ever had a pelvic exam^e^	193 (94.1)	53 (86.9)	36 (92.3)	104 (99.0)
Ever had a Pap test^d^	187 (92.1)	48 (80.0)	35 (92.1)	104 (99.0)
Had a Pap test in the last 3 years^e^	157 (76.6)	38 (62.3)	32 (82.1)	87 (82.9)

a Missing data are excluded from totals.

b *P* < .05, one-way ANOVA for association between literacy level and characteristic.

c Results are not significant.

d *P* < .001, chi-square test of association between literacy level and characteristic.

e *P* < .01, chi-square test of association between literacy level and characteristic.

f *P* < .05, chi-square test of association between literacy level and characteristic.

**Table 2:** Bivariate and Logistic Regression Models of Having a Papanicolaou (Pap) Test Ever and Within the Last Three Years: Survey Results Among Latinas Aged 40 and Older (n = 205), New York City, November 2002–July 2003^a^

	**Bivariate**		**Adjusted**	

	**Had Recent Pap (<3 years)**	**Ever Had Pap**	**Had Recent Pap (<3 years)**	**Ever Had Pap**

**Source of care**				

No source of care	Ref	Ref	Ref	Ref
Has source of care	2.82 (1.46–5.44)	0.81 (0.29–2.29)	3.67 (1.45–9.27)	0.83 (0.19–3.54)

** Health insurance status**				

No insurance	Ref	Ref	Ref	Ref
Private, Medicaid and/or Medicare	1.74 (0.89–3.43)	0.85 (0.32–2.31)	0.63 (0.23–1.73)	0.98 (0.22–4.44)

**Age (years)**				

40–49	Ref	Ref	Ref	Ref
50–59	0.52 (0.24–1.10)	0.77 (0.23–2.55)	0.46 (0.19–1.13)	1.38 (0.34–5.55)
60 and older	0.44 (0.19–0.99)	0.56 (0.17–1.86)	0.41 (0.15–1.07)	1.34 (0.33–5.42)

**Years in United States**				

Less than 15	Ref	Ref	Ref	Ref
15 or more	2.05 (1.06–3.97)	1.46 (0.53–3.99)	2.05 (0.94–4.47)	1.20 (0.36–4.06)

**Education**				

Elementary or no schooling	Ref	Ref	Ref	Ref
Some high school or more	1.62 (0.85–3.10)	5.24 (1.46–18.82)	1.03 (0.44–2.44)	3.16 (0.60–16.66)

**Health Literacy Level**				

Adequate	Ref	Ref	Ref	Ref
Marginal	0.95 (0.36–2.48)	0.11 (0.01–1.11)	1.31 (0.44–3.85)	0.14 (0.01–1.41)
Inadequate	0.34 (0.17–0.71)	0.04 (0.005–0.30)	0.53 (0.21–1.35)	0.06 (0.01–0.55)

a Values represent odds ratios (95% confidence intervals). Ref indicates referent group.
